# Chronic Atypical Myelomonocytic Leukemia of Eosinophilic Lineage in a Cat With Feline Leukemia Virus: A First Case Report

**DOI:** 10.1155/crve/6729159

**Published:** 2026-02-26

**Authors:** Morales Orozco Pablo José, Magaña Anzaldo Aida Evelyn, Bernal Muñoz Diana Betsabe, Saavedra Ruvalcaba Adolfo Giovanni, Rosales Camacho Paulina

**Affiliations:** ^1^ Hematologia veterinaria GDL, Guadalajara, Jalisco, Mexico

**Keywords:** cytochemistry, eosinophilic, feline leukemia virus

## Abstract

Feline hematopoietic neoplasms associated with retroviruses pose a diagnostic and therapeutic challenge, especially when they present with atypical morphological features that do not fit conventional classifications. This work describes the first reported case worldwide of atypical chronic myelomonocytic leukemia of eosinophilic lineage in a cat positive for feline leukemia virus, diagnosed through detailed cytomorphological analysis and the use of enzymatic cytochemical stains. The rarity of this case underscores the importance of documenting and studying uncommon presentations, with the goal of expanding clinical knowledge and strengthening diagnostic tools in feline veterinary medicine.

## 1. Introduction

Myeloproliferative and myelodysplastic neoplasms are a heterogeneous group of hematopoietic malignancies characterized by dysplasia and marrow proliferation. These features overlap with laboratory and morphological characteristics of various neoplasms in both blood and bone marrow ([1]).

Chronic myelomonocytic leukemia (CMML) is a clonal malignant hematopoietic neoplasm characterized by hybrid features of myeloproliferative disorder and myelodysplastic disorder, as recognized by the World Health Organization (WHO) [2].

In this case, we report the first case of a chronic atypical myelomonocytic leukemia of eosinophilic lineage in a cat secondary to feline leukemia virus (FeLV).

## 2. Case Presentation

On October 16, 2024, a bone marrow aspirate was requested by the hematology service for a 2‐year‐old cat that was presented at another veterinary center with severe dyspnea, weakness, pallor, anorexia, and weight loss over 2 weeks.

On physical examination prior to sampling, the patient was cachectic and pale, with a temperature of 38.6°C, a heart rate of 150 bpm, a respiratory rate of 70 rpm, a mild cardiac murmur, poor coat condition, and slight splenomegaly. No lymphadenopathy or apparent skin masses were observed.

A complete blood count (CBC) was performed at an external veterinary center, revealing severe hematological alterations, such as white blood cells (45.09 × 10^9^/L), monocytes (12.5 × 10^9^/L), eosinophils (15.00 × 10^9^/L), lymphocytes (4.35 × 10^9^/L), neutrophils (13.15 × 10^9^/L), red blood cells (1.75 × 10^12^/L), hemoglobin (4.56 g/dL), packed cell volume (13%), and platelets (17 × 10^9^/L).

The cat was diagnosed as positive for FeLV using the SNAP Feline Triple Test by IDEXX Laboratories Inc., Westbrook, Maine, United States.

### 2.1. Diagnostic and Complementary Tests

Based on the clinical history provided by the primary veterinarian and the previously conducted blood work, an immediate bone marrow aspirate was decided upon. The procedure was performed with a 14‐G Rosenthal needle and a 5‐mL plastic syringe coated with a small amount of EDTA as an anticoagulant.

The patient was lightly sedated with dexmedetomidine at 0.4 mcg/kg and ketamine at 2 mg/kg injected via a 1‐mL syringe with a removable needle through a 24‐G silicone catheter placed in the right cephalic vein.

Once anesthesia was induced, the left shoulder was shaved, and the area was aseptically cleaned. Local anesthesia was administered subcutaneously with lidocaine (2%) at 6 mg until reaching the periosteum of the greater tuberosity of the humerus.

After waiting 5 min post‐lidocaine administration, the Rosenthal needle was inserted through the same skin puncture until contacting the greater tuberosity. Oscillations of 180° were made until penetrating the bone and reaching the medullary cavity. After entering the bone marrow, the 5‐mL lubricated syringe was attached, and the plunger was retracted to collect 1 mL of bone marrow. This sample was deposited into an EDTA tube for 5 mL of blood, mixed to homogenize, and prevented from coagulating.

From the liquid bone marrow sample, 25 smears of 4.8 *μ*L each were made on glass slides.

**Figure 1:**
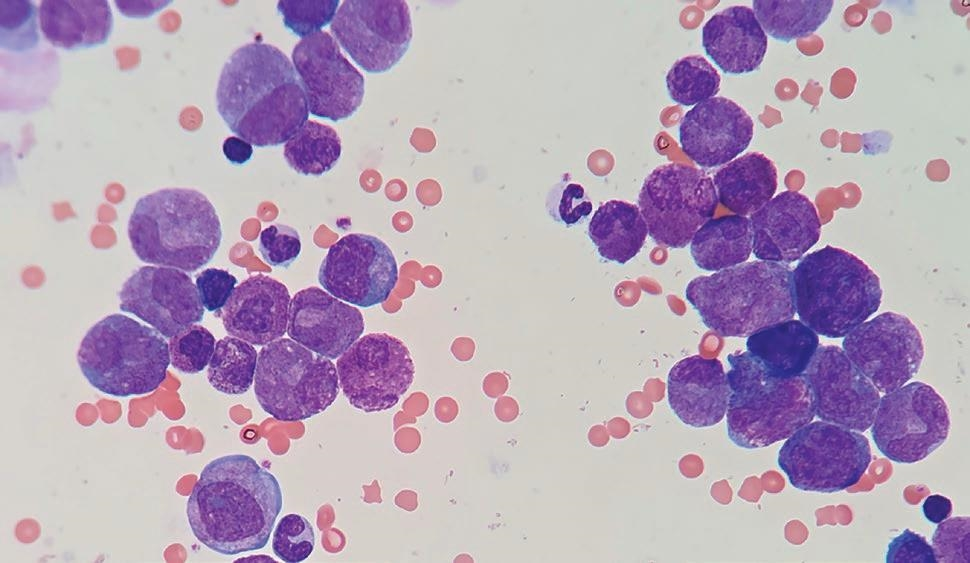
Wright‐stained sample of bone marrow revealing severe dysplastic changes.

**Figure 2:**
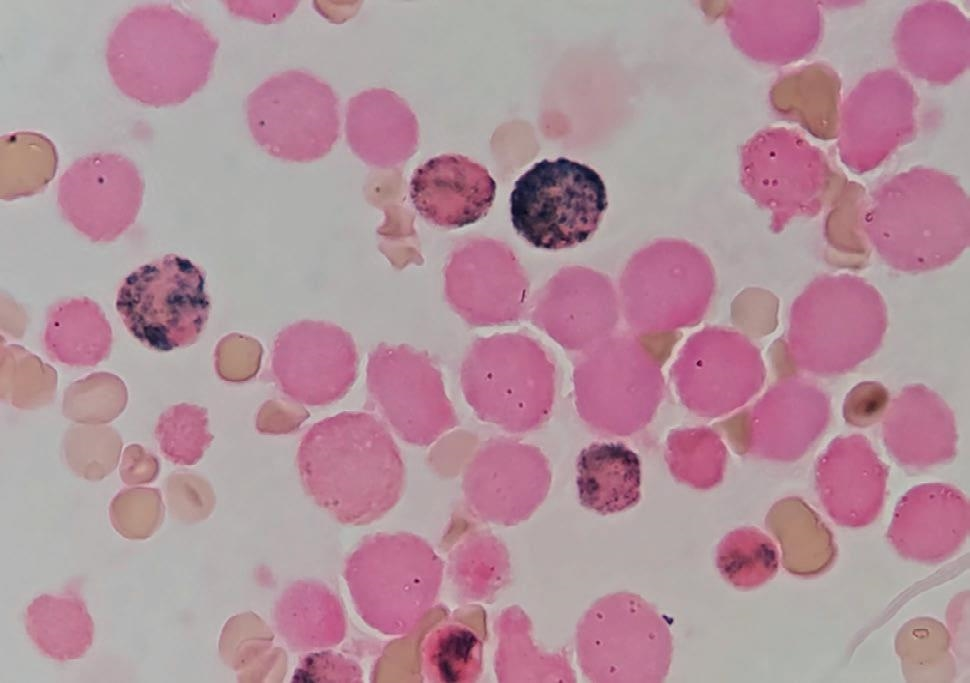
Enzymatic cytochemical stain for myeloperoxidase showing negativity for most of the cells except some mature neutrophils.

**Figure 3:**
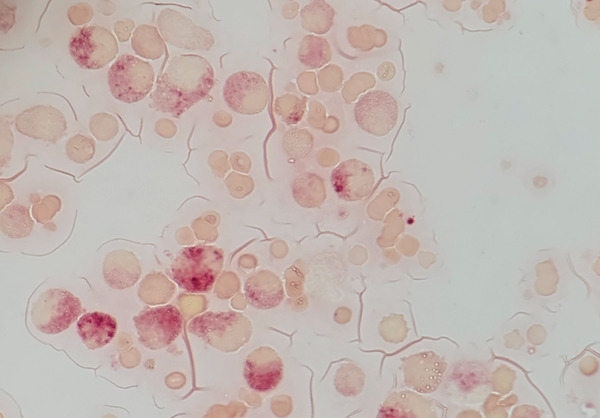
Enzymatic cytochemical stain alpha‐naphthyl acetate esterase, revealing positivity for most of the cells morphologically compatible with neutrophilic lineage but instead being of monocytic lineage.

These smears were processed with various stains: five with Wright stain at pH 6.8, as shown in Figure 1; three with myeloperoxidase via enzymatic cytochemistry, as shown in Figure 2; three with alpha‐naphthyl acetate esterase via enzymatic cytochemistry, as shown in Figure 3; and three with Perl′s stain for nonheme iron detection with no relevant findings.

A differential count of 1000 cells was performed on Wright‐stained slides. A marked increase in cells of evident eosinophilic morphology and mature monocytes was observed in all maturation stages. A marked increase in dysplastic changes, characterized principally by gigantic mature eosinophils, hypersegmented eosinophils, gigantic eosinophilic metamyelocytes, and abnormal mitotic figures, was also observed. Apparently, the neutrophilic lineage was within normal ranges.

Enzymatic cytochemical stains for myeloperoxidase and alpha‐naphthyl acetate esterase were performed to corroborate the lineage of the cells previously described, revealing that most of the cells that were morphologically compatible with myeloid/neutrophilic lineage were, in reality, of monocytic lineage.

Based on the findings obtained from bone marrow examination along with peripheral blood studies, a diagnosis of atypical CMML of the eosinophilic lineage is concluded. This is in accordance with the criteria described by Onida and Chalandon [1] (white blood cell count greater than 13 × 10^9^/L, monocyte count over ≥0.5 × 10^9^/L, and exceeding 10% of the total counted leukocytes, with less than 20% blasts in both blood and bone marrow, and not fulfilling criteria for other hematopoietic neoplasms).

Atypical observation includes 45.4% of medullary cells belonging to the eosinophilic lineage (morphologically), which are negative for myeloperoxidase and alpha‐naphthyl acetate esterase by enzymatic cytochemical methods. Then, 21.3% of cells that were morphologically compatible with myeloid/neutrophilic lineage were positive in a diffuse pattern to alpha‐naphthyl acetate esterase. This indicates severe morphological dysplasia characterized mainly by giant cells, aberrant nuclei, abnormal mitoses, hyposegmented nuclei, and giant harlequin cells (Figure 1). Under nonpathological conditions, it is acceptable to observe up to 15% of cells belonging to the eosinophilic lineage in the bone marrow without any form of dysplasia.

## 3. Discussion and Conclusions

According to the WHO diagnostic criteria, it involves persistent monocytosis in peripheral blood without observed blast counts exceeding 20% in blood or marrow, along with morphological dysplastic changes in the observed cells [2].

It should be emphasized that in this specific case, hypereosinophilia was observed in both blood and marrow, which causes this patient′s classification to not fit the classic WHO criteria and should be considered atypical.

It is known that cats positive for FeLV or feline immunodeficiency virus (FIV) have a higher incidence of hematopoietic neoplasms, mainly lymphoma [3].

The clinical course of patients with myeloproliferative disorders and myelodysplastic syndromes can range from indolent over years to severe, progressing to acute leukemia in humans [1].

Patients with myeloproliferative and myelodysplastic neoplasms should be evaluated for multiple chromosomal changes associated with various diseases [4]. In this case, karyotyping was not possible because the patient died 2 days after diagnosis.

As demonstrated in their article [2], enzymatic cytochemistry stains are useful to differentiate cell lines in cases of severe dysplasia.

In conclusion, hematopoietic neoplasms are more commonly observed in feline patients with retroviral diseases, which can predispose them to the development of rare neoplastic processes.

It is important to address these cases promptly and perform relevant tests to begin diagnosing these pathologies, understand them more in‐depth, and potentially employ appropriate therapies in the future.

According to the conducted research, this is the first worldwide reported case of atypical eosinophilic CMML in a feline patient and the first case in Mexico where a hematological malignancy was diagnosed using enzymatic cytochemical stains.

## Funding

No funding was received for this manuscript.

## Consent

No written consent has been obtained from the patients, as there is no patient‐identifiable data included in this case report.

## Conflicts of Interest

The authors declare no conflicts of interest.

## Data Availability

Data sharing is not applicable to this article as no datasets were generated or analyzed during the current study.
